# Comment on: Climate change is a health issue. The general practitioner and planetary health by Stoffers & Muris 2023

**DOI:** 10.1080/13814788.2023.2298332

**Published:** 2024-01-24

**Authors:** Oisín Brady Bates, Natasha Freeman

**Affiliations:** a Public Health and Primary Care, IPH, Trinity College Dublin Faculty of Health Sciences, Dublin, Ireland; b Royal College of General Practitioners, London, UK

As representatives of the European Young Doctors Special Interest Group in Planetary Health, we would like to respond to a joint editorial by Jelle Stoffers and Jean Muris concerning the important role of general practice in responding to the urgent planetary health crisis [1]. The authors have kindly referenced our Planetary Health Repository as a source of freely available information on Planetary Health for GPs. We want to express our gratitude for mentioning our work and respond in agreement with the points that they raise.

Our Planetary Health Special Interest Group collects a wide range of documents, links, and literature relevant to planetary health to form our Repository. We felt that there was already so much information available on this subject that it would now be useful to consolidate all of it into a central location that was free to use and widely shared. We hope that the app and database-style format will help busy health professionals access relevant information quickly and easily, and thus be more likely to integrate planetary health into their own practice. We are committed to continuously improving and expanding our Repository based on user feedback. To this end, we would appreciate users trying it out and suggesting any feedback or resources they would consider useful to include, through our user survey. All this can be accessed *via* our website [2].

A couple of other significant resources that may be of interest to readers (and which can be found in the Repository) include the recently published ‘Glas toolkit’ (Glas = green in the English language), which outlines sustainable practice-based actions and activities tailored to Irish general practice [3]. There is also a free international planetary health for primary care massive open online course available in English which is hosted by TelessaúdeRS-Federal University of Rio Grande do Sul (UFRGS) [4].

We welcome an increasing focus on addressing planetary health within general practice in Europe. General practice/family medicine is ideally situated to tackle the health issues arising from climate change and worsening environmental instability.
